# Objective Measurement of 24-Hour Movement Behaviors in Preschool Children Using Wrist-Worn and Thigh-Worn Accelerometers

**DOI:** 10.3390/ijerph18189482

**Published:** 2021-09-08

**Authors:** Marieke De Craemer, Marga Decraene, Iris Willems, Feija Buysse, Ellen Van Driessche, Vera Verbestel

**Affiliations:** 1 Department of Rehabilitation Sciences, Ghent University, 9000 Ghent, Belgium; Marga.Decraene@UGent.be (M.D.); willems.iris@UGent.be (I.W.); Vera.Verbestel@UGent.be (V.V.); 2 Research Foundation Flanders, 1000 Brussels, Belgium; 3 Department of Movement and Sports Sciences, Ghent University, 9000 Ghent, Belgium; 4 Department of Public Health and Primary Care, Ghent University, 9000 Ghent, Belgium; Feija.buysse@UGent.be (F.B.); ellenvandriessche10@hotmail.com (E.V.D.)

**Keywords:** ActivPAL, Axivity, preschoolers, 24-h movement behaviors, physical activity, sitting time, sleep duration, objective measurement

## Abstract

In recent years, more attention has been paid towards the study of 24-h movement behaviors (including physical activity (PA), sedentary behavior (SB) and sleep) in preschoolers instead of studying these behaviors in isolation. This study aimed to evaluate the feasibility of using wrist- vs. thigh-worn accelerometers and to report accelerometer-derived metrics of 24-h movement behaviors in preschoolers. A convenience sample of 16 preschoolers (50.0% boys, 4.35 years) and one of their parents were recruited for this study. Preschoolers had to wear the ActivPAL accelerometer (attached to the upper thigh) and Axivity accelerometer (using a wrist band) simultaneously for 7 consecutive days and for 24 h a day. Parents completed an acceptability survey. In total, 16 preschoolers (100.0%) had a minimum of 6 days of valid wrist-worn data, while only 10 preschoolers (62.5%) had a minimum of 6 days of valid thigh-worn data (*p* = 0.002). When looking at the acceptability, 81.3% of parents indicated that it was easy for their child to wear the Axivity for 7 consecutive days, and 93.8% of parents indicated so for the ActivPAL (*p* = 0.88). However, some parents stated that the wristband of the Axivity accelerometer was big, which might have affected data collection. Significant differences were found for the measurement of total volume of PA, SB and sleep across 24 h. Total PA was 464.44 min/day (±64.00) with the ActivPAL compared with 354.94 min/day (±57.46) with the Axivity (*p* < 0.001). The volume of SB was 290.94 min/day (±55.05) with the ActivPAL compared with 440.50 min/day (±50.01) with the Axivity (*p* < 0.001). The total volume of sleep was also significantly different between both devices (*p* = 0.001; ActivPAL: 684.63 min/day ± 51.96; Axivity: 645.69 min/day ± 46.78). Overall, parents perceived both devices to be feasible to use for preschoolers. However, future studies are required to validate both devices for the measurement of preschoolers’ 24-h movement behaviors since significant differences in the classification of PA, SB and sleep were found in this small sample.

## 1. Introduction

To promote healthy growth and development in children, the World Health Organization (WHO) introduced the “24-h movement behavior guidelines” for the early years (0–5 years old) in 2019 [1]. The WHO guidelines state that preschoolers (3–5 years old) should (1) engage in physical activity (PA) for a minimum of 180 min per day at any intensity, of which 60 min should be at moderate to vigorous intensity, (2) limit screen time (i.e., using screens such as television, computer, tablet) to a maximum of 60 min per day and limit periods of uninterrupted sitting to a maximum of 60 min at the time and (3) have a good quality sleep between 10 to 13 h per night [1]. These guidelines are highly innovative as they no longer focus on one particular behavior but integrate all behaviors that are conducted within a 24-h time span, namely PA, sedentary behavior (i.e., sitting time) (SB) and sleep. These behaviors interact, which means that time spent on one of these behaviors (e.g., PA) has an effect on the time that can be spent on (one of) the other behavior(s) (e.g., SB and/or sleep) [2]. Sleep, SB and PA are distributed across a 24-h time span on a movement continuum going from no movement (sleep, SB) to high movement (PA). Complying with the 24-h movement behavior guidelines in preschoolers is already associated with health-related quality of life, social-cognitive development and behavioral and emotional problems in this young age group [3]. This underlines the importance of taking all movement behaviors across a 24-h time span into account, since it is already associated with desirable health indicators at a young age.

The current 24-h movement behavior guidelines for the early years incorporate screen time as contextual SB but do not take total daily sitting time into account. Given the fact that PA, SB and sleep are interrelated within one day, it might be interesting to have a better understanding of the distribution of time spent in these behaviors across 24 h. At the moment, there are no studies yet looking at objectively measured 24-h movement behaviors in preschoolers. Although several measurement devices already exist and are being used, most studies on 24-h movement behaviors in preschool children use a combination of objective (e.g., accelerometers) and subjective methods (e.g., proxy-reported questionnaires) [4,5,6,7].

However, subjective measurement methods are accompanied by several disadvantages. Because preschoolers do not yet have the cognitive ability to self-report their daily levels of PA, SB and sleep, parents report on their child’s behavior [8,9,10]. It is, however, impossible for parents to constantly monitor their child, since preschoolers also spend their time in absence of their parents, at preschool, for example [8]. 

To the best of our knowledge, there are currently no studies looking at measurement devices that can objectively measure preschoolers’ 24-h movement behaviors. One of the research gaps cited by the WHO was “to examine the entire 24-h day and PA, SB and sleep duration in young children”, illustrating the importance of a measurement device that is able to objectively quantify preschoolers’ PA, SB and sleep [1]. The aim of this pilot study was therefore to investigate the parent-reported feasibility of using the ActivPAL accelerometer and the Axivity accelerometer for several days with regard to measuring preschoolers’ 24-h movement behaviors, and to compare the volumes of PA, SB and sleep from both measurement devices.

## 2. Materials and Methods

### 2.1. Study Protocol

An observational, cross-sectional study was conducted in which convenience and snowball sampling was used to recruit parents and their three- to five-year-old child in West and East Flanders, Belgium. A call through social media, preschools and after school childcare centers was used to recruit parents and their children. Parents provided an active informed consent prior to data collection. This study was approved by the Ethics Committee of the Ghent University Hospital (2019/1394).

Originally, data collection was planned between November 2019 and the end of March 2020. Unfortunately, recruitment of participants and data collection were prematurely ended due to the COVID-19 pandemic and the complete lockdown in Belgium starting on Friday the 13th of March 2020. Therefore, measurements were conducted between November 2019 and February 2020. Participants were visited at home by one of three researchers (FB, EVD, MDC) to explain the aim of the study and to mount the measurement devices. The children had to wear two measurement devices (ActivPAL and Axivity accelerometers) during a full weekend. Both measurement devices were collected after the measurement period had ended.

### 2.2. Measurements

#### 2.2.1. ActivPAL Accelerometer

The ActivPAL accelerometer (PAL Technologies Ltd., Glasgow, UK) is a small (53 × 35 × 7 mm), lightweight (20 g), unobtrusive measurement device that consists of both an accelerometer (MEMS, uniaxial) and an inclinometer. The device was initialized using ActivPAL software (version 7) before use. After the ActivPAL was made waterproof by covering the device with a thin finger cot and a transparent 3M Tegaderm tape as proposed by the manufacturer, it was mounted on the preschooler’s right anterior thigh midway between the hip and knee, using a second transparent and hypoallergenic 3M Tegaderm tape (10 × 10 cm). The ActivPAL had to be worn 24 h per day for seven consecutive days. Parents were given additional tapes and an instruction letter how to replace the tape themselves if necessary.

#### 2.2.2. Axivity Accelerometer

The Axivity (AX3; Axivity Ltd., Newcastle, United Kingdom) is a data logger including a MEMS 3-axis accelerometer which enables the device to collect movement data. It is also small (23 × 32.5 × 7.6 mm), lightweight (11 g) and unobtrusive, and can be worn around the wrist. Before the start of data collection, the Axivity was synchronized and initialized to start measuring simultaneously with the ActivPAL accelerometer using the Open Movement software (OMGui, version 1.0.0.43; Newcastle University, Newcastle, UK). Preschoolers were fitted with the Axivity (simultaneously with the ActivPAL) using a wrist band at the non-dominant wrist. Parents were instructed to only remove the device for water-based activities such as showering or swimming.

#### 2.2.3. Short Questionnaire

Parents reported on their child’s age, sex, ethnicity, height and weight. Height and weight were used to calculate children’s Body Mass Index (BMI; weight/height in m²). Based on age- and sex-specific cut-offs [11], preschoolers’ weight status (normal weight, overweight, obese) was determined. Parents were asked whether their child found it pleasant or unpleasant to wear the measurement devices using two different questions: “How did the child experience wearing the wrist-worn accelerometer” (i.e., the Axivity), and “How did the child experience wearing the thigh-worn accelerometer” (i.e., the ActivPAL). A 5-point scale (ranging from highly pleasant to highly unpleasant) was used to indicate whether their child found it pleasant or unpleasant to wear the measurement devices. In addition, two questions were asked whether parents found it feasible to let their child wear the devices: “How did you perceive the feasibility of the wrist-worn accelerometer?” (i.e., the Axivity), and “How did you perceive the feasibility of the thigh-worn accelerometer?” (i.e., the ActivPAL). A 3-point scale was used to understand whether it was feasible to wear the devices for seven consecutive days (yes, feasible for all 7 days; yes, feasible for a couple of days; no, not feasible).

### 2.3. Data Reduction and Analysis

Next to feasibility information about wearing both devices, data from both devices were analyzed. To be included in the analyses, concurrent data of the ActivPAL and Axivity had to be available for at least 24 consecutive hours. One 24-h timeframe was randomly selected from the data. Every randomly selected 24-h timeframe started at 7:00 a.m. in the morning until 6:59 a.m. the day after if data of both devices were available.

#### 2.3.1. ActivPAL Accelerometer

Minute-by-minute ActivPAL data were downloaded using the ActivPAL-software. The VANE algorithm available in the ActivPAL-software was used to identify sitting, standing and stepping. In addition, the CREA algorithm available in the ActivPAL-software was used to identify the “time in bed”. Both algorithms provided separate CSV-files which were combined into one CSV-file to match the time in bed with the time the child was awake. For every minute of data, the number of seconds spend sedentary, standing or stepping was provided. Then, one full 24-h time block was randomly selected. A similar protocol as in the study of De Decker et al. (2013) was used to obtain a binary classification for each 60 s interval to enable comparison with the Axivity data [12]. If more than 45 s of the 60 s epoch were spent sitting or lying, this epoch was classified as SB (1) in the SB column. If less than 45 s of the 60 s epoch were spent sitting or lying, this epoch was not classified as SB (0) in the SB column. Based on the CREA algorithm, the time in bed (including start time and end time) was classified as sleeping (1). A 0 (not sleeping) was added for time spent out of bed. In the activity column, a 0 was added if there was a 1 in only one of the columns of SB or sleeping. A 1 was noted down when there was a 0 in the SB column AND in the sleeping column. In the end, the sum of all minutes in all three columns was made to reflect the total amount of minutes spend in PA, SB or sleep across a full 24-h day when using an ActivPAL accelerometer.

#### 2.3.2. Axivity Accelerometer

Axivity data were downloaded and processed using the Open Movement software (OMGui). The result was an Excel file in which every minute was coded as either SB or PA. The same 24-h block of data as from the ActivPAL-data were selected from the Axivity data. There were four different columns in the Excel file, one column for sitting, one column for light PA, one column for moderate PA and one column for vigorous PA. By means of a 0 (=no) or a 1 (=yes), it was reflected for each minute whether the child engaged in SB, light PA, moderate PA or vigorous PA. In addition, a second Excel file was obtained through the Open Movement software in which all periods of preschoolers’ sleep were reflected minute-by-minute, and this was added to the Excel file with the periods of SB and PA. In the end, the sum of all minutes was again made to reflect the total amount of daily minutes spend in PA, SB or sleep across a full 24-h day when using an Axivity accelerometer.

### 2.4. Data Analysis

Analyses were performed using SPSS version 25. Descriptive statistics (%) were used to describe the sample characteristics, to describe the feasibility of both measurement devices and to express the percentage of time spent in PA, SB and sleep as measured by the ActivPAL and Axivity accelerometer. Paired sample t-tests were performed to analyze the difference in mean minutes of sleep, PA and SB across 24 h measured by the ActivPAL on the one hand and the mean minutes of sleep, PA and SB across 24 h by the Axivity on the other hand.

## 3. Results

### 3.1. Descriptive Characteristics of the Sample

In total, parents of 17 preschoolers provided consent to participate in the study. Of those 17 preschool children, 16 (94.1%) provided valid data (i.e., data on 24 consecutive hours by both measurement devices) that could be analyzed (50.0% boys, mean age: 4.35 (±0.72) years). All preschool children were of Belgian origin. Ten preschool children (83.4%) had a normal weight, two preschool children had overweight (12.5%), three children were underweight (18.8%) and data on height and weight were not reported by the parents of one child (6.3%).

**Figure 1:**
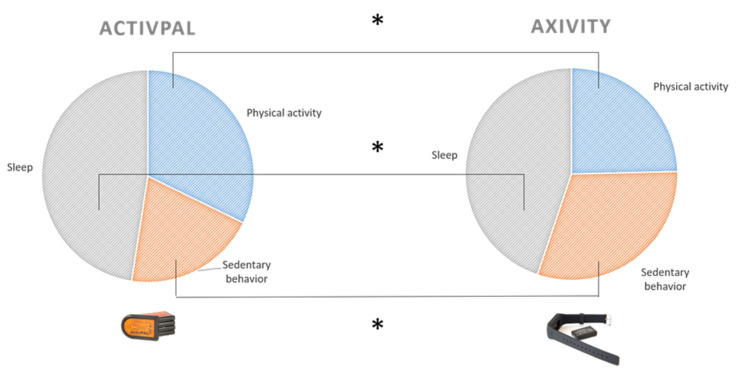
Visual representation of the distribution of time in physical activity, sedentary behavior and sleep across 24 h measured with the ActivPAL on the one hand and the Axivity on the other hand. * Significant difference between ActivPAL and Axivity accelerometer (*p* < 0.01).

In total, 16 preschoolers (100.0%) had a minimum of 6 days of valid wrist-worn data, while only 10 preschoolers (62.5%) had a minimum of 6 days of valid thigh-worn data (*p* = 0.002). The minimum of days with valid data using the ActivPAL was two days (*n* = 1), and the maximum was seven days (*n* = 5). Table 1 and Figure 1 show the percentage of time spent in objectively measured PA, SB and sleep measured with the ActivPAL and the Axivity accelerometer. Paired sample t-tests showed a significant difference in the measurement of PA (t = 6.45, *p* < 0.001) with the ActivPAL measuring a higher mean volume of PA minutes across 24 h (x = 464.44 min/day; standard deviation (SD = 64.00) compared with the Axivity (x = 354.94 min/day; SD = 57.46). Another significant difference was found for the measurement of SB (t = −11.16, *p* < 0.001) with the ActivPAL measuring a lower volume of SB across 24 h (x= 290.94 min/day; SD = 55.05) compared with the Axivity (x = 440.50 min/day; SD = 50.01). A significant difference was also found for the measurement of sleep with the ActivPAL measuring a higher volume of sleep compared with the Axivity (t = 4.13, *p* = 0.001; ActivPAL: x = 684.63 min/day; SD = 51.96; Axivity: x = 645.69 min/day; SD = 46.78) (Table 1).

### 3.2. Feasibility of Using the ActivPAL and Axivity Accelerometers in Preschool Children

Regarding the ActivPAL, nine parents (56.3%) indicated that their preschool child found it pleasant to highly pleasant to wear the ActivPAL. Six parents (37.5%) were neutral and one parent (6.3%) indicated that their child found it unpleasant to wear the device. Seven parents (43.8%) indicated on a 5-point scale that their preschool child found it pleasant to highly pleasant to wear the Axivity. Eight parents (50%) were neutral, one parent (6.3%) indicated ‘other’ and wrote down that the Axivity was too big for the small wrists of their preschooler, causing the Axivity to move back and forth which made it less pleasant to wear.

In total, 15 parents (93.8%) found it feasible to let their child wear the ActivPAL for seven consecutive days, and one parent (6.3%) found it feasible to let their child wear the ActivPAL for a couple of days. Regarding the Axivity, 13 parents (81.3%) found it feasible to let their child wear the Axivity for seven consecutive days, two parents (12.5%) found it feasible to let their child wear the device for a couple of days and one parent (6.3%) marked ‘other’ and wrote down the problem of the wrist band being too big.

## 4. Discussion

The current study was a pilot study with the aim to assess the feasibility of using the ActivPAL and the Axivity accelerometer for several days in this young age group based upon parents’ opinions, and to compare these two objective measurement devices in measuring PA, SB and sleep across 24 h in preschoolers.

First of all, both accelerometers were perceived by almost 50% of preschoolers as pleasant to wear. However, this means that almost half of preschoolers perceived wearing the accelerometers as neutral and even one child found it unpleasant to wear the ActivPAL. Furthermore, most parents found it feasible to let their child wear both devices for seven consecutive days. These results are remarkable, since the percentage of parents making this statement about the ActivPAL accelerometer is much higher (93.8%) compared with the percentage of children actually having valid data for seven days (31.3%). In addition, one parent reported that the standard wrist band of the Axivity was too large for the child’s wrist, making it less pleasant to wear the device. Since data collection took place in winter, most parents used their child’s thick winter clothing as a tool to decrease the mobility of the wristband by mounting the wristband on top of their child’s sweater, jumper or cardigan. However, using the Axivity with the standard wrist bands might cause problems regarding data collection in warmer months (spring, summer) since children are wearing light clothing which might cause more movement of the wrist band and, respectively, more noise in the data collection. Other studies in young children measuring, e.g., PA with the Axivity accelerometer [13] used a specifically designed fabric band around the wrist which enables the measurement of PA in young children without the problem of having a wrist band which is too big.

Although reported in other studies looking at practicality and feasibility in using the ActivPAL accelerometer in preschool children, parents did not make any notifications about skin irritation in the current study. In the study of De Decker et al. (2013), 38% of preschool children had skin irritation due to the tape used to attach the device to the upper thigh. The study of Davies et al. (2012) only made recordings of one child with skin irritation, but the protocol in the study of Davies et al. (2012) was different since the device was taken off for water-based activities, which makes it impossible to use their proposed protocol to measure 24-h movement behaviors [14]. Although not in preschool children but in 11- to 18-year-old adolescents, the study of Shi et al. (2019) also showed a high percentage of skin irritation when using the ActivPAL, which eventually resulted in taking off the ActivPAL [15]. The study of Shi et al. (2019) also required the adolescents to continuously wear the device, which was comparable with our study protocol [15].

The ActivPAL and the Axivity accelerometer were compared using minute-by-minute data of both devices. The major finding of those analysis was that there was a considerable difference between the ActivPAL and the Axivity accelerometer concerning the measurement of all three behaviors. The mean time in either PA and SB as measured by the ActivPAL and the Axivity was largely different with a mean difference ranging from 110 and 150 min, which corresponds to 2 to 2.5 h. If you take into account that the PA guidelines state that preschoolers should be physically active for 180 min per day, it is apparent that the choice of measurement device has a large influence on the results that will be reported regarding preschoolers’ compliance with the guidelines. Although the results for sleep duration seem more promising with a smaller mean difference (i.e., around 40 min), still, a significant difference was found between both measurement devices. In addition, a difference of 40 min of sleep also has a large impact when investigating the prevalence of sleep duration or the proportion of preschool children complying with the sleep duration guidelines. Our results therefore show that there is a large difference in the measurement of PA, SB and sleep between both monitors. When choosing the ActivPAL in measuring preschoolers’ 24-h movement behaviors, researchers should be aware that the volume of PA will be higher compared with using the Axivity, while the volume of SB will be lower compared with using the Axivity. Researchers need to take their research question and the characteristics of the measurement device into account before selecting the measurement device relevant for their specific studies. The fact that one parent noticed that the wrist band of the Axivity was too large for the child’s wrist might indicate that the preschool age might be too early to use the Axivity using the current available accessories of the device (i.e., wrist band), unless the wrist band created and produced by Open Lab (Newcastle, UK) and used in the study of Prioreschi et al. (2017) in infants and toddlers can be used. In addition, the large wrist band might be a possible reason for the difference in measuring PA and SB, since possible shifts of the device across the wrist might have caused noise in the data. Future studies should therefore take the placement of the accelerometer into consideration with the aim to optimally answer the research question of the study.

Compared with validation studies of measurement devices [12,16], more research on the validation of the ActivPAL and the Axivity accelerometer to measure 24-h movement behaviors is needed. In fact, the original aim of the study was to validate both ActivPAL and Axivity accelerometers to measure 24-h movement behaviors by using direct observation since direct observation can be seen as the golden standard in measuring behaviors. The goal was to film 24-h in a preschooler’s day using a GoPro camera. Unfortunately, several limitations appeared after data collection. These were limited battery life and storage capacity causing data loss. In addition, there was an inability to track the starting point of the recording due to a reset of the camera settings every time the camera was switched off. The study of Willets et al. (2018) used a Vicon Autographer wearable camera during waking hours [17]. This kind of wearable camera has the advantage of having a long battery life (~16 h), and storage capacity for over one week’s worth of images [18]. The camera automatically takes photographs every 20 s and each photograph is time-stamped which means that duration of activities (e.g., sitting, cycling) can be captured [18]. However, due to preschoolers’ intermittent movement behavior pattern [19] it might be a challenge to use this type of wearable camera in this age group, since different activities can be performed within a timeframe of 20 s. This might mean that different short bouts of activity or sitting time might be missed using this kind of camera. Another possibility might be to use several fixed cameras on a tripod that are posted in different corners of the room to ensure that the activities and postures of the child are caught on video. However, again due to preschoolers’ intermittent pattern of behaviors [19], this might be challenging since preschoolers’ might leave the room with the cameras as well. In addition, it causes an additional burden for preschoolers’ parents. Moreover, it might be possible to use live streaming cameras with an automatic upload in the cloud which enables the fact that we exactly know at what time the direct observation started and problems with memory capacity are avoided.

Although we are aware that this is a pilot study and data should be interpreted with caution, larger scaled studies (e.g., >100 participants) are necessary to have a clear and more generalized view on preschoolers’ 24-h movement behaviors. In addition, larger scaled validation studies (e.g., >30 participants) using, e.g., a Vicon Autographer wearable camera as golden standard might reveal the device (i.e., ActivPAL or Axivity) that is the most valid to measure preschoolers’ 24-h movement behaviors. Information from this kind of validation study in combination with parents’ opinions about the feasibility of using these measurement devices might provide the best knowledge to advance the measurement of preschoolers’ 24-h movement behaviors.

A strength of the current study is the fact that this is the first study that compared two objective measurement devices towards measuring 24-h movement behaviors and their feasibility to use them in an extremely young age group. The largest limitation is the small sample due to the fact that COVID-19 appeared during data collection, but also the protocol was intensive in combination with the direct observation (24 h of filming), which induces the fact that the results should be interpreted carefully.

## 5. Conclusions

Overall, parents perceived both devices to be feasible to use for preschoolers. There is a difference in measuring PA, SB and sleep using the ActivPAL on the one hand and the Axivity on the other hand. The ActivPAL measures a higher volume of PA across 24 h and a lower volume of SB across 24 h compared with the Axivity. The current study is a pilot study, and future studies should look at incorporating a golden standard such as direct observation to validate both measurement devices for the measurement of PA, SB and sleep across 24 h.

## Figures and Tables

**Table 1:** Descriptive statistics of 24-hour movement behaviors measured by the ActivPAL and Axivity accelerometers for the total sample (*n* = 16 participants).

	ActivPAL Accelerometer			Axivity Accelerometer			Mean Difference
	% of the Day	Mean ± SD	Min–Max	% of the Day	Mean ± SD	Min–Max	
Volume of physical activity/24 h	32.3	464.44 ± 64.00	335–590	24.6	354.94 ± 57.46	235–454	109.50 *
Volume of sedentary behavior/24 h	20.2	290.94 ± 55.05	162–358	30.6	440.50 ± 50.01	317–496	−149.56 *
Volume of sleep/24 h	47.5	684.63 ± 51.96	589–788	44.8	645.69 ± 46.78	543–735	38.94 *

* Significant difference between ActivPAL and Axivity accelerometer (*p* < 0.01).

## Data Availability

Data sharing is applicable upon request.

## References

[1] World Health Organization Guidelines on Physical Activity, Sedentary Behaviour and Sleep for Children under 5 Years of Age. https://apps.who.int/iris/handle/10665/311664.

[2] Chaput J.P., Carson V., Gray C.E., Tremblay M.S. (2014). Importance of all movement behaviors in a 24 h period for overall health. Int. J. Environ. Res. Public Health.

[3] Rollo S., Antsygina O., Tremblay M.S. (2020). The whole day matters: Understanding 24-hour movement-guideline adherence and relationships with health indicators across the lifespan. J. Sport Health Sci..

[4] De Craemer M., McGregor D., Androutsos O., Manios Y., Cardon G. (2018). Compliance with 24-hour movement behaviour guidelines among belgian pre-school children: The ToyBox-Study. Int. J. Environ. Res. Public Health.

[5] Chen B., Bernard J.Y., Padmapriya N., Yao J., Goh C., Tan K.H., Yap F., Chong Y.S., Shek L., Godfrey K.M. (2019). Socio-demographic and maternal predictors of adherence to 24-hour movement guidelines in Singaporean children. Int. J. Behav. Nutr. Phys. Act..

[6] Chaput J.P., Colley R.C., Aubert S., Carson V., Janssen I., Roberts K.C., Tremblay M.S. (2017). Proportion of preschool-aged children meeting the Canadian 24-Hour Movement Guidelines and associations with adiposity: Results from the Canadian Health Measures Survey. BMC Public Health.

[7] Cliff D.P., McNeill J., Vella S.A., Howard S.J., Santos R., Batterham M., Melhuish E., Okely A.D., de Rosnay M. (2017). Adherence to 24-Hour Movement Guidelines for the Early Years and associations with social-cognitive development among Australian preschool children. BMC Public Health.

[8] Corder K., van Sluijs E.M., Wright A., Whincup P., Wareham N.J., Ekelund U. (2009). Is it possible to assess free-living physical activity and energy expenditure in young people by self-report?. Am. J. Clin. Nutr..

[9] Ekelund U., Tomkinson G., Armstrong N. (2011). What proportion of youth are physically active? Measurement issues, levels and recent time trends. Br. J. Sports Med..

[10] Pate R.R., O’Neill J.R., Mitchell J. (2010). Measurement of physical activity in preschool children. Med. Sci. Sports Exerc..

[11] Cole T.J., Lobstein T. (2012). Extended international (IOTF) body mass index cut-offs for thinness, overweight and obesity. Pediatr. Obes..

[12] De Decker E., De Craemer M., Santos-Lozano A., Van Cauwenberghe E., De Bourdeaudhuij I., Cardon G. (2013). Validity of the ActivPAL and the ActiGraph monitors in preschoolers. Med. Sci. Sports Exerc..

[13] Prioreschi A., Brage S., Hesketh K.D., Hnatiuk J., Westgate K., Micklesfield L.K. (2017). Describing objectively measured physical activity levels, patterns, and correlates in a cross sectional sample of infants and toddlers from South Africa. Int. J. Behav. Nutr. Phys. Act..

[14] Davies G., Reilly J.J., McGowan A.J., Dall P.M., Granat M.H., Paton J.Y. (2012). Validity, practical utility, and reliability of the activPAL in preschool children. Med. Sci. Sports Exerc..

[15] Shi Y., Huang W.Y., Yu J.J., Sheridan S., Sit C.H., Wong S.H. (2019). Compliance and Practical Utility of Continuous Wearing of activPAL in Adolescents. Pediatr. Exerc. Sci..

[16] Janssen X., Cliff D.P., Reilly J.J., Hinkley T., Jones R.A., Batterham M., Ekelund U., Brage S., Okely A.D. (2014). Validation of activPAL defined sedentary time and breaks in sedentary time in 4- to 6-year-olds. Pediatr. Exerc. Sci..

[17] Willetts M., Hollowell S., Aslett L., Holmes C., Doherty A. (2018). Statistical machine learning of sleep and physical activity phenotypes from sensor data in 96,220 UK Biobank participants. Sci. Rep..

[18] Doherty A.R., Hodges S.E., King A.C., Smeaton A.F., Berry E., Moulin C.J., Lindley S., Kelly P., Foster C. (2013). Wearable cameras in health: The state of the art and future possibilities. Am. J. Prev. Med..

[19] Cliff D.P., Reilly J.J., Okely A.D. (2009). Methodological considerations in using accelerometers to assess habitual physical activity in children aged 0–5 years. J. Sci. Med. Sport.

